# Molecular Dynamics Simulations Reveal the HIV-1 Vpu Transmembrane Protein to Form Stable Pentamers

**DOI:** 10.1371/journal.pone.0079779

**Published:** 2013-11-06

**Authors:** Siladitya Padhi, Nabab Khan, Shahid Jameel, U. Deva Priyakumar

**Affiliations:** 1 Centre for Computational Natural Sciences and Bioinformatics, International Institute of Information Technology, Hyderabad, India; 2 International Centre for Genetic Engineering and Biotechnology, New Delhi, India; University of Technology Sydney, Australia

## Abstract

The human immunodeficiency virus type I (HIV-1) Vpu protein is 81 residues long and has two cytoplasmic and one transmembrane (TM) helical domains. The TM domain oligomerizes to form a monovalent cation selective ion channel and facilitates viral release from host cells. Exactly how many TM domains oligomerize to form the pore is still not understood, with experimental studies indicating the existence of a variety of oligomerization states. In this study, molecular dynamics (MD) simulations were performed to investigate the propensity of the Vpu TM domain to exist in tetrameric, pentameric, and hexameric forms. Starting with an idealized α-helical representation of the TM domain, a thorough search for the possible orientations of the monomer units within each oligomeric form was carried out using replica-exchange MD simulations in an implicit membrane environment. Extensive simulations in a fully hydrated lipid bilayer environment on representative structures obtained from the above approach showed the pentamer to be the most stable oligomeric state, with interhelical van der Waals interactions being critical for stability of the pentamer. Atomic details of the factors responsible for stable pentamer structures are presented. The structural features of the pentamer models are consistent with existing experimental information on the ion channel activity, existence of a kink around the Ile17, and the location of tetherin binding residues. Ser23 is proposed to play an important role in ion channel activity of Vpu and possibly in virus propagation.

## Introduction

The human immunodeficiency virus type-1 (HIV-1) employs a range of viral proteins to successfully establish and propagate infection in the host. These include the structural envelope (Env gp120, gp41), capsid (p24^CA^) and matrix (p17^MA^) proteins, the enzymes reverse transcriptase, ribonuclease H, integrase and protease, two regulatory proteins (Rev, Tat), and four accessory proteins (Nef, Vif, Vpr and Vpu) [1]. Of these, the accessory proteins are not required for viral replication in vitro but are indispensable for the establishment and persistence of HIV infection and pathogenesis [2]. The viral protein U (Vpu) is an 81-amino acid transmembrane (TM) protein encoded by HIV-1 that increases virus release from host cells [3,4]. The protein, however, is not encoded by the less virulent human immunodeficiency virus type-2 (HIV-2) and simian immunodeficiency virus (SIV) [4]. Knowledge of the three-dimensional structure of the oligomeric form of Vpu is expected to further our understanding of its functional mechanisms, and this can possibly be exploited as a drug target [5-7].

Vpu is a type I integral membrane protein with an N-terminal TM domain and a C-terminal cytoplasmic domain [8]. The cytoplasmic domain contains two alpha helices [9,10] and is involved in the degradation of CD4 molecules at the endoplasmic reticulum [11,12]. Between the alpha helical domains are two serine residues, Ser52 and Ser56 [13], which must necessarily be phosphorylated for Vpu to exhibit its CD4 degradation activity [14]. The TM domain helps in viral release from host cells [12], which is brought about by the degradation of tetherin, an antiviral protein encoded by host cells that causes retention of virions on the cell surface [15]. Three residues in the Vpu TM domain, Ala14, Ala18, and Trp22, have been shown to be important for this activity [16]. The ability of Vpu to oligomerize [8] allows it to form cation-selective ion channels [17]. Such channels can form both in planar lipid bilayers and in the plasma membrane of *Escherichia coli* in vivo [17], and they are known to facilitate viral release [5]. The ion channel activity ascribed to the TM domain of Vpu [5] appears to be rather weak and the characteristics of the channel almost resemble those of a pore [18]. Gel permeation chromatography studies show Vpu to be a pentamer [19], but recent photo-induced cross-linking studies indicate that a variety of oligomeric states might exist [20].

Solution NMR studies in lipid micelles on Vpu_2-37_, a truncated form of Vpu containing the N-terminal TM domain, showed an α-helix spanning residues 9 to 29 [21]. Similar studies on Vpu_2-30_, a peptide containing residues 2 to 30 from Vpu with a 6-residue solubility tag, revealed an α-helix spanning residues 8 to 25 [22]. The helix has a kink around Ile17, and is tilted at an angle of 13° with respect to the membrane. Fourier transform infrared (FTIR) spectroscopy on the first 31 N-terminal residues of Vpu indicate an α-helix with a tilt of (6.5±1.7)° and a rotational pitch angle of (283±11)° around Val13 [23]. Simulated annealing with restrained molecular dynamics on rotationally symmetrical tetramers, pentamers, and hexamers of Vpu shows that only a pentamer has a rotational pitch angle for Val13 close to the experimental value [23]. Molecular dynamics (MD) simulations restraining the motion of ions to the axis of the pore show that the conductance of the pentamer is closer to the experimentally observed value than either the tetramer or the hexamer [24]. MD simulations performed using an octane layer for mimicking the properties of a lipid bilayer have shown that a helix is expelled from a hexameric arrangement; the same was not observed for a pentamer [25,26]. A number of modeling and simulation studies have been carried out by modeling the channel as a homo-pentamer [27-32]. Pentamer models have also been generated using pre-equilibrated monomers and these show the lumen of the pore to be a hydrophobic stretch [32]. However, all of the studies mentioned above were carried out with the assumption that the native oligomeric state is a pentamer. Notably, the modeling studies that originally suggested Vpu to form a pentamer did not take into consideration a fully hydrated lipid bilayer environment [24-26]. A systematic study of the Vpu TM domain tetramers, pentamers and hexamers, taking into account the explicit lipid environment would provide atomistic details on the oligomeric structure, and the factors that determine the stability of the native structure.

A useful approach for evaluating the stability of different oligomeric states of a membrane protein is the use of replica-exchange molecular dynamics (REX/MD) in an implicit membrane environment [33,34]. REX/MD overcomes the problem of entrapment in local minima [35], thereby making the sampling of regions of phase space possible that are otherwise not accessible to constant temperature molecular dynamics. Implicit membrane models take into consideration the physical environment in which a membrane protein finds itself without having real membrane and solvent molecules, making the approach computationally efficient [33,36]. Combining implicit membrane models with REX/MD has made it possible to estimate the stability of different oligomeric states in terms of both potential energy and free energy [33,34]. This study was carried out to model the possible oligomeric states of the Vpu TM domain, and to understand the structural and energetic factors that make one oligomeric state more stable than others. Briefly, REX/MD simulations have been used with an implicit membrane model for sampling varied conformations of different possible oligomeric states of Vpu. Representative structures have then been selected for more extensive studies in fully hydrated lipid bilayers. The pentameric state is shown to be the most favored state, and structural features of the protein are described that help explain its function.

## Results and Discussion

### Higher oligomers display reduced tilt

**Figure 1:**
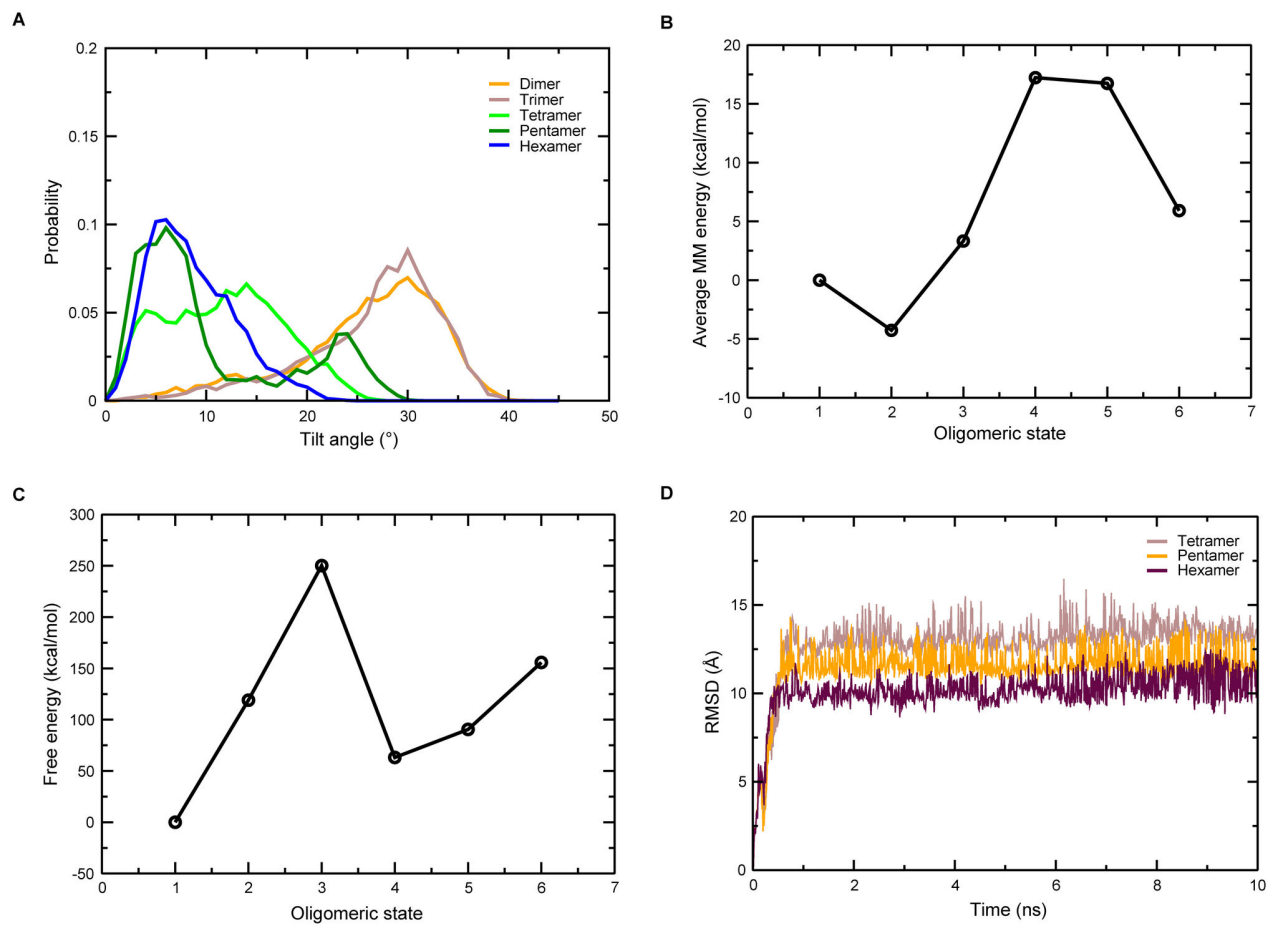
Replica-exchange molecular dynamics in an implicit membrane environment. (A) Probability distribution of the tilt angle for the conformations sampled at 300 K from the last 9 ns of replica-exchange molecular dynamics. (B) Average potential energy and (C) free energy of the different oligomeric states over the last 9 ns of replica-exchange molecular dynamics. The values shown are relative to the monomer. (D) RMSD of the tetramer, the pentamer, and the hexamer in the REX/MD simulations.

The implicit membrane model that was used for the REX/MD simulations uses generalized Born electrostatics for modelling the solvent on both sides of the membrane [36]. The membrane hydrophobic core is represented using a low-dielectric slab with a fixed thickness [33]. Thus, when there is mismatch between the hydrophobic regions of a helical protein and the continuum solvent region, the membrane cannot respond by altering its thickness. The only mechanism by which such a mismatch is minimized is by tilting of the helices to ensure that as much of the hydrophobic part of the protein lies in the membrane as possible. Figure 1A shows the probability distribution of the tilt angles of different oligomers over the last 9 ns of REX/MD. The tilt of the helices was seen to decrease with an increase in the number of helices in the system. In the higher oligomers (tetramer, pentamer and hexamer), the helices are packed closely together, allowing interhelical interactions to occur. These interactions are stabilizing, so the helices are able to overcome the destabilizing effect of hydrophobic mismatch. Thus, the protein does not have to tilt too much to attain an energetically stable conformation. In the dimer and trimer, however, interhelical interactions are almost absent with hydrophobic mismatch being the major factor affecting the orientation of helices, and the protein tilts until most of its hydrophobic residues are buried in the membrane core.

### Tetramer, pentamer, and hexamer are possible oligomeric states

The average molecular mechanical potential energy of the TM domain in different oligomeric states relative to the monomeric state is shown in Figure 1B. The energies of all the states were comparable, with the average energy of any given oligomeric form differing by less than 17 kcal/mol compared to the monomeric form. Based on this analysis, no particular oligomer could be identified as the native form. Bu et al. have suggested the necessity of considering entropic factors due to the assembly of helices [34]. The entropy loss accompanied by the formation of a given oligomeric state was calculated by taking the entropy term for the monomer as the reference. The free energy values (Table 1 and Figure 1C), which were obtained after taking into account entropy loss and stabilization arising from solvation, indicate the tetramer and pentamer to be stable oligomeric states. There is an increase in the free energy from monomer to dimer, and from dimer to trimer; this is followed by a remarkable decrease in the free energy in the trimer-tetramer transition, and a slight increase in going from tetramer to pentamer to hexamer. Figure 1D shows the RMSD of the tetramer, the pentamer, and the hexamer in the REX/MD simulations. The RMSD changes rapidly in the initial period before being converged, indicating rearrangement of the helices until a stable conformation is attained. It is important to note that the helices in the initial conformation were placed at large separations to encourage rotation of the helices (Figure S1 in File S1).

**Table 1:** Entropy loss and free energies of the different oligomeric states in the replica-exchange MD simulations.

**Oligomeric state**	**Potential energy (kcal/mol)**	**Solvation energy (kcal/mol)**	**Rotational entropy term, TS_rot_ (kcal/mol)**	**Translational entropy term, TS_trans_ (kcal/mol)**	**Vibrational entropy term, TS_vib_ (kcal/mol)**	**Free energy (kcal/mol)**
Monomer	487.32	-295.42	0	0	0	191.90
Dimer	483.06	-195.19	1.81	0.62	-25.46	310.90
Trimer	490.65	-108.26	2.34	0.98	-63.03	442.10
Tetramer	504.56	-232.64	1.86	1.24	13.79	255.03
Pentamer	504.07	-207.78	2.16	1.44	10.29	282.40
Hexamer	493.24	-152.71	2.42	1.60	-11.15	347.66

**Figure 2:**
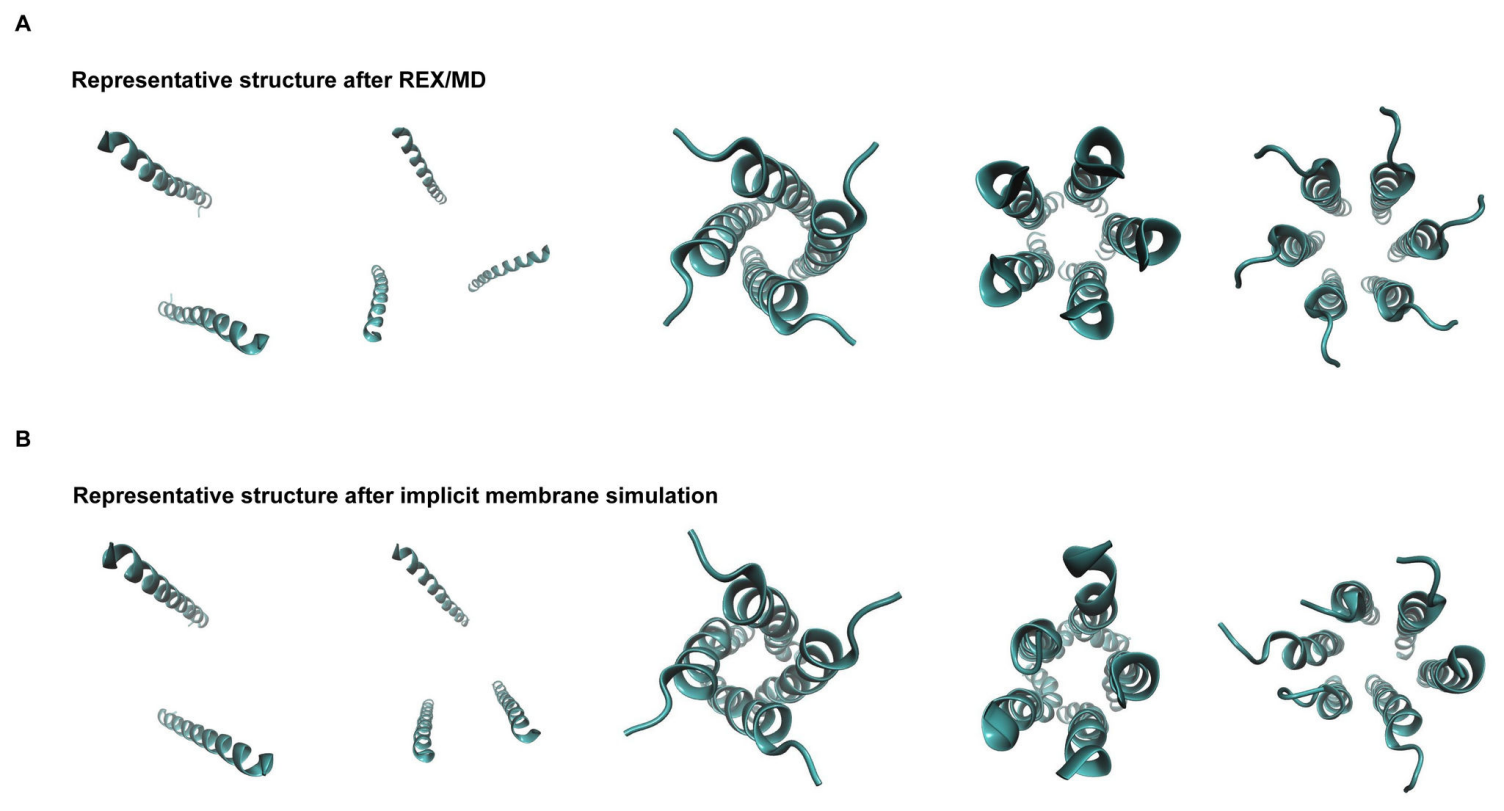
Representative structures. The structures for the different oligomeric states before (top row) and after (bottom row) 10 ns simulation in an implicit membrane environment are shown.

Equilibration of the representative structures sampled from REX/MD showed that the dimer and trimer do not form a compact structure, with the helices lying far apart (Figure 2). Since the dimer and trimer are not feasible oligomeric structures, the rest of this report is concerned with the tetramer, pentamer and hexamer, unless stated otherwise. The use of REX/MD ensures that much of the phase space of the various oligomeric states, and a majority of all possible conformations are sampled. As shown above, the tetramer and pentamer were identified as stable oligomeric states using REX/MD with an implicit membrane. Although it is able to model the physical characteristics of the membrane hydrophobic core and bulk solvent, a drawback of the generalized Born implicit membrane model used here is that it does not take into account the hydrophilic nature of the pore region, which is central to the functioning of ion channels and strongly influences the orientation and behavior of residues lining the pore. A more realistic representation of the channel is therefore possible only with accurate modeling of the pore region.Thus, a comprehensive investigation of the stabilities of the tetramer, the pentamer, and the hexamer has been carried out in a hydrated lipid bilayer environment.

### Explicit membrane MD simulations reveal the pentamer to be the most stable oligomeric state

**Figure 3:**
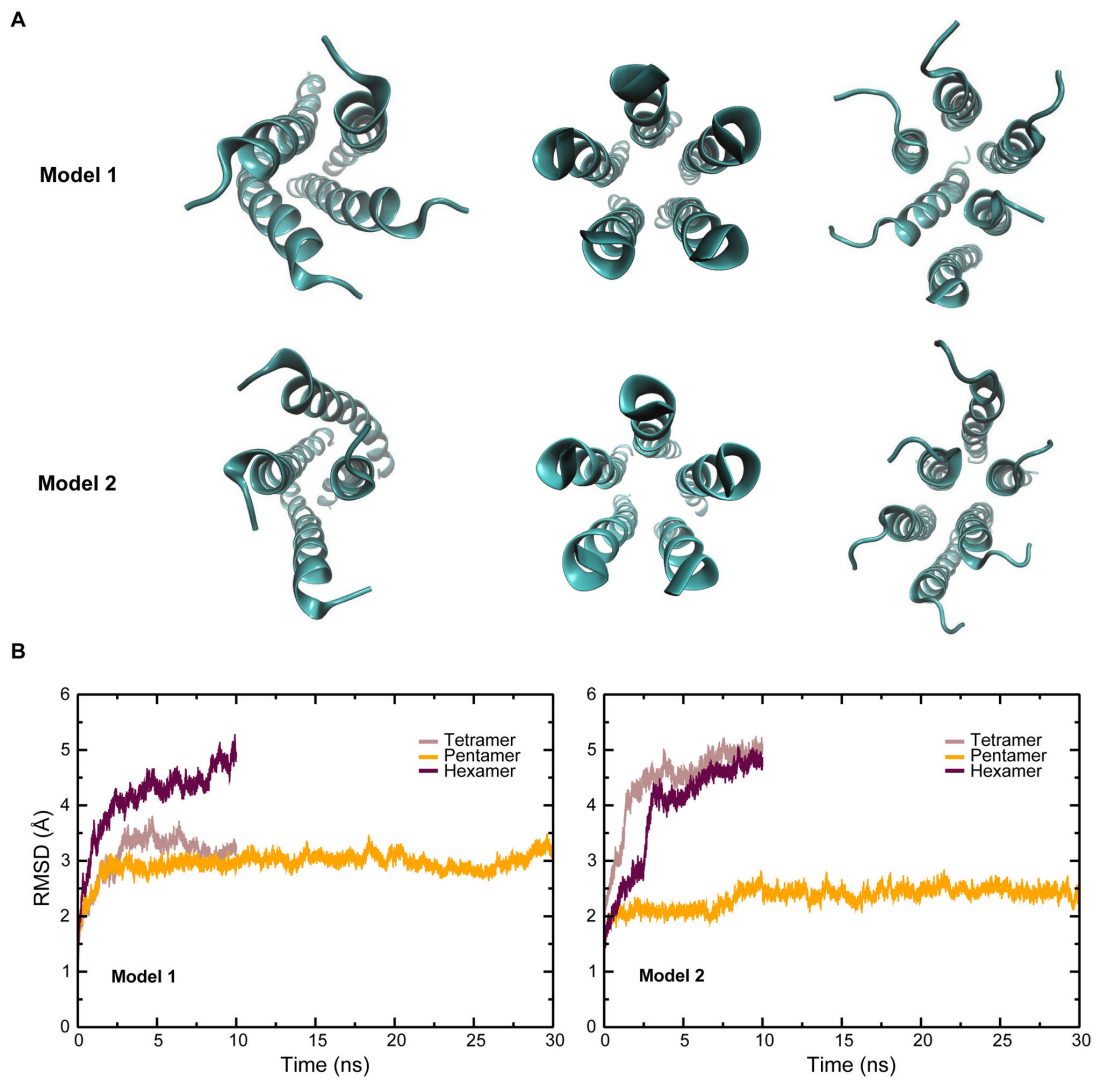
Molecular dynamics in an explicit membrane environment. (A) Models for the tetramer, pentamer and hexamer after simulation in a fully hydrated lipid bilayer. The images for the pentamer are after 30 ns, and those for the tetramer and hexamer are after 10 ns. The lipid bilayer and solvent molecules have been omitted for clarity. The pentamer retained a channel-like structure in both the simulations. (B) RMSD of the different oligomeric states.

Two independent simulations were carried out for each of the tetrameric, pentameric and hexameric states in the explicit bilayer environment (see Methods section). In both sets of simulations, visual inspection revealed that only the pentamer retained the rotational symmetry necessary for forming an ion channel (Figure 3A). Using root mean-square deviation (RMSD) values, the pentameric forms were observed to attain equilibration in the first 2 ns adapting itself to the membrane environment, but the tetramers and the hexamers become distorted and completely lose their initial pore-like structure (Figure 3B). Such distortion was seen in both the model structures for the tetrameric and hexameric states. However, model 1 of the tetramer exhibits RMSD values that are comparable to the pentamer. Interestingly, in one of the hexamer models (Model 1), a helix is expelled from the bundle, leading to a pentameric structure. Such expulsion has been reported by Lopez et al. in studies on a hexameric form of the Vpu TM domain in an octane environment [26], and is indicative of a propensity of the Vpu TM domain to exist in a pentameric state. This is in agreement with earlier studies which suggest that the oligomer exists in a pentameric form [19,23]. The possible factors that favor the pentameric form over the other forms are elucidated below.

### The helices in the pentamer are held together by strong van der Waals interactions

**Figure 4:**
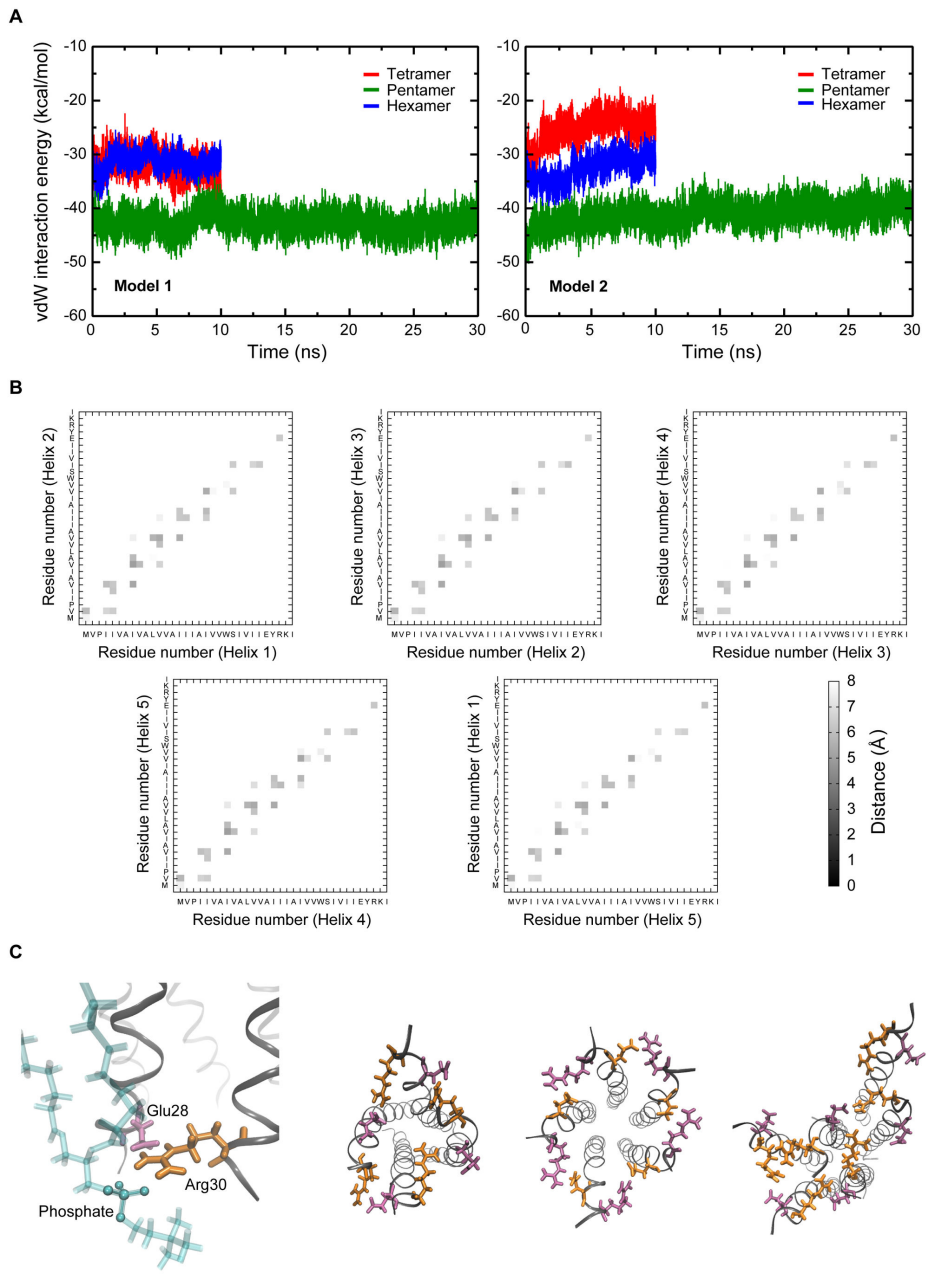
Interhelical interactions in the oligomers. (A) Interhelical van der Waals interaction energy per helix pair for the tetramer, the pentamer, and the hexamer in a lipid bilayer environment. The interhelical van der Waals interaction energy was calculated for all adjoining helix pairs in the oligomer and then divided by the number of helices to give the average value per helix pair. (B) Contacts between residues on adjoining helices. Residue-residue distances have been averaged over time. The values shown are for Model 1. (C) Orientation of Arg30 (“licorice” representation, colored orange). One of the amino groups forms a salt bridge with Glu28 (“licorice” representation, colored mauve) on a neighboring helix, while the other interacts with headgroup oxygens (headgroup phosphate is shown in “CPK” representation). The TM domain is shown in “ribbons” representation, and a POPC molecule is shown in “bonds” representation. The five salt bridges in the tetramer, pentamer and hexamer are also shown.

We looked at the van der Waals interaction energy between neighbouring helices in the different oligomers. As can be seen clearly, interhelical van der Waals forces greatly stabilize the helices in the pentamer (Figure 4A and Table S1 in File S1). The interhelical distances and rotational symmetry of the pentameric state are probably better suited for van der Waals interactions than other oligomeric states. The significance of van der Waals interactions over other forms of nonbonded interactions between adjoining helices can be attributed to the fact that the residues in the interface between adjoining helices are non-polar, as apparent from the contact maps shown in Figure 4B (contact maps for model 2 are shown in Figure S4A in File S1). Since the structural features of models 1 and 2 are similar, only the figures for model 1 are shown hereafter, with images for model 2 shown in File S1. The non-polar residues form close contacts, making effective interhelical interactions. Most interhelical contacts occur on the N-terminal side of the channel, where the residues are all hydrophobic. A notable exception to the non-polar nature of these contacts is a salt bridge between Glu28 and Arg30 on neighboring helices, which is seen in the top right corner of the contact maps (Figure 4B and Figure S4A in File S1). One of the amino groups on the Arg30 side chain faces the carboxyl group on Glu28, while the other amino group points towards a phosphate oxygen in a nearby lipid molecule as shown in model 1 (Figure 4C and Figure S4B in File S1, panel on extreme left). The salt bridge is seen to occur between all pairs of neighboring helices (Figure 4C and Figure S4B in File S1), and it exists consistently in both the simulations. Such a salt bridge satisfies the hydrogen bond requirements for the two charged residues, thereby stabilizing the residues in an otherwise hydrophobic environment. In the tetramer and hexamer, however, the salt bridge is absent in some pairs of adjacent helices; this might be an important factor in stabilizing the oligomer.

**Figure 5:**
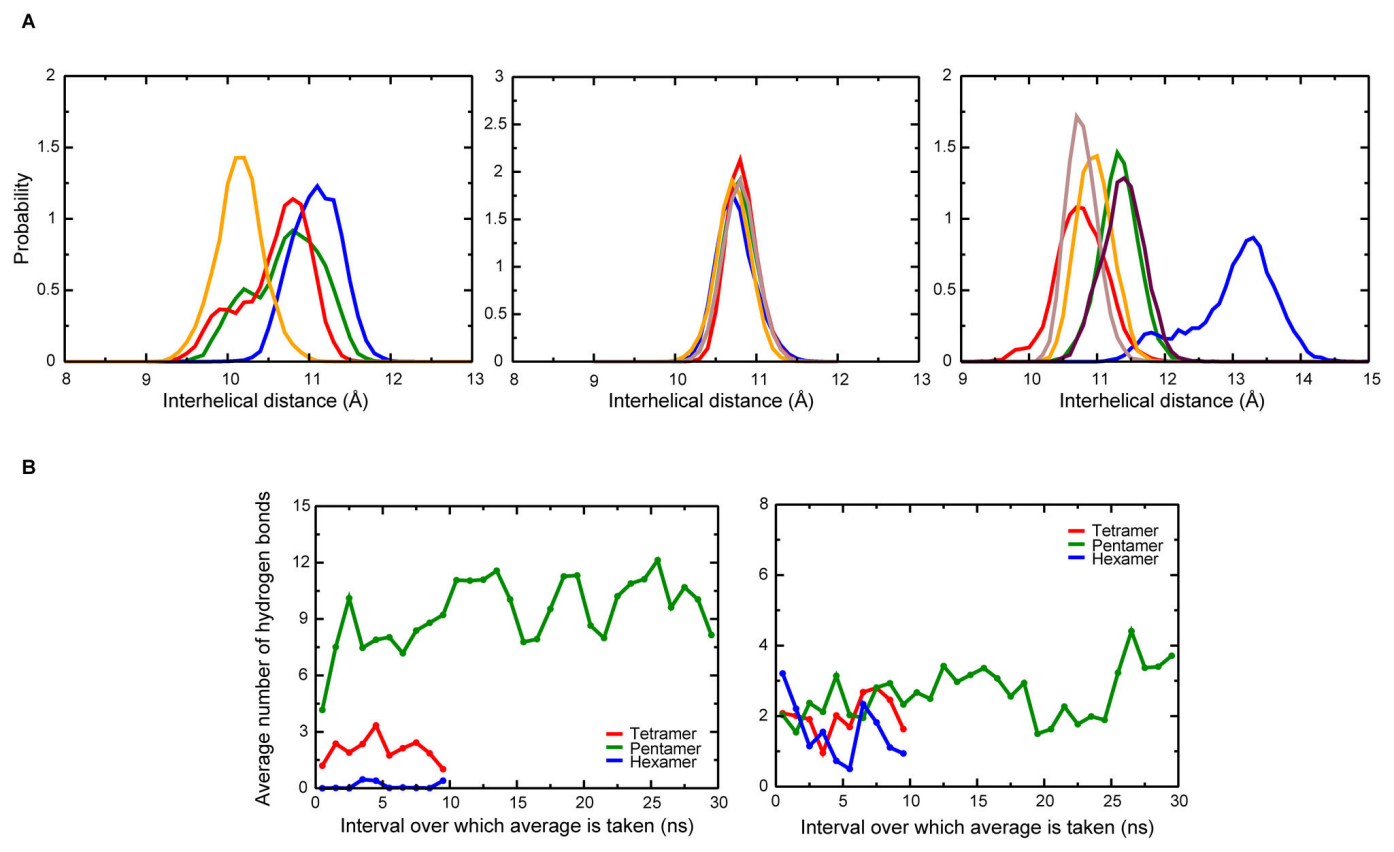
Interhelical distance and protein-lipid interactions. (A) Probability distribution of interhelical distance for tetramer, pentamer and hexamer. The distance between the centres-of-mass of adjoining helices was calculated. Only the helical backbone was considered, and the top three and bottom three residues were neglected. (B) Average number of hydrogen bonds between lipid headgroups and polar residues for Arg30 and headgroup (left panel), and Tyr29 and headgroup (right panel). The cutoffs used were 3.5 Å for the donor-acceptor distance, and 45° for the donor-hydrogen-acceptor angle.

The probability distribution for interhelical distances between all adjoining helix pairs over the simulation was carried out, with the distance between the centres-of-mass of adjacent helices taken as the interhelical distance. The terminal residues were not considered in calculating the centre-of-mass to avoid high fluctuations due to these residues. The narrow distribution for the pentamer suggests that the interhelical distance remains stabilised at a given distance (Figure 5A and Figure S3A in File S1). Furthermore, the occurrence of peaks centered around the same point for all helix pairs in the pentamer indicates that the interhelical distance is almost the same in all helix pairs. However, for the tetrameric and hexameric structures, the interhelical distances calculated for the helical pairs and the broad nature of some curves indicate structures that are not so stable (Figure 5A and Figure S3A in File S1). These observations support highly symmetric nature of the pentameric structure, but not the tetrameric or hexameric structures. 

### The hydrophilic and basic residues in the TM domain interact with lipid headgroups

We then estimated the average number of hydrogen bonds between polar residues on the protein and the lipid headgroups (the cutoffs used in calculating the number of hydrogen bonds were 3.5 Å for the donor-acceptor distance, and 45° for the donor-hydrogen-acceptor angle). As described above, the arginine residues within the TM domain have one of their amino groups facing the lipid headgroup, allowing the formation of hydrogen bonds between the side chain and the headgroup oxygen atoms. The number of such interactions with the headgroup is significantly higher in the pentamer (Figure 5B and Figure S3B in File S1), and might play a role in adhering the protein to the lipid bilayer. Tyrosine residues are also able to form hydrogen bonds, although the number of bonds is fewer in number than those due to arginine, especially in the pentamer. This might be due to tyrosine side chains lying slightly above the plane of the headgroup oxygens, while the arginine side chains lie in the plane of these oxygens. Moreover, the arginine side chains are oriented in a direction perpendicular to the axis of the lipid molecules, thereby making favorable hydrogen bond angles. Interactions between positively charged residues and lipid headgroups have previously been shown to be crucial in structures of ion channels as reported by both experimental [37,38] and computational studies [39,40].

### A hydrophobic region occurs around the middle of the channel

**Figure 6:**
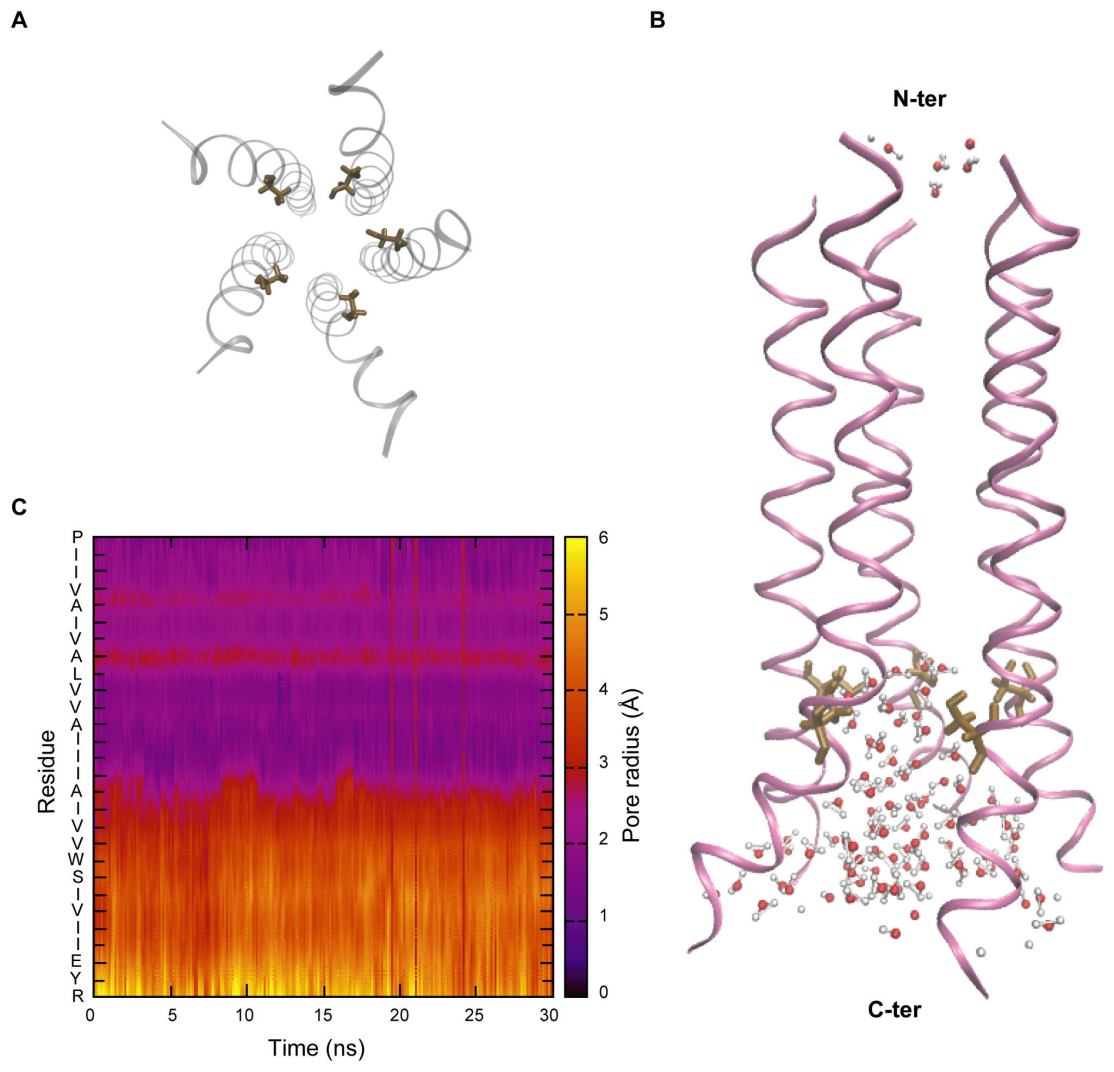
Pore profile. (A) View along the pore axis from the C-terminal showing the Ser23 residue in “licorice” representation. Serine faces the interior of the channel in the pentamer model. (B) Side view of the pentamer model showing the location of the Ser23 residue (in “licorice” representation) and water molecules in the pore. The N-terminal side is on the top and the C-terminal is at the bottom. (C) Pore radius across the axis of the pentamer model. The pore is constricted towards the N-terminal side (top half).

The Ser23 residue faces the interior of the channel (Figure 6A and Figure S5A in File S1), providing a hydrophilic region in the pore around that residue. Notably, the initial orientations of the side chains were chosen to be random in which Ser23 was facing the exterior of the pore in the beginning of the REX/MD simulations (see Figure S1 in File S1). The explicit lipid bilayer simulations were started with a conformation where the pore was uniformly solvated, but most of the water molecules were expelled within the first 50 ps of the equilibration period. At the end of 30 ns of production run, much of the pore water was concentrated around the serine residue and towards the ends of the pore, near bulk water, rather than being spread uniformly across the pore (Figure 6B and Figure S5B in File S1). This is because the residues occurring in the middle of the protein are all hydrophobic, and the part of the channel lined by these residues, consequently, has a predominantly hydrophobic environment. Furthermore, the pore is constricted towards the N-terminal, leaving less room for the accommodation of water molecules. As seen in a dynamical variation mapping of the pore radius, the narrowest part of the pore occurs in the middle around Val12 and Ile16 (Figure 6C and Figure S5C in File S1). The occurrence of hydrophobic residues along this narrow stretch is likely to impose an energy barrier to the transport of ions, and this region might play a role in controlling the kinetics of ion conduction. It is possible that the channel is in a closed conformation, since a large part of the pore is devoid of water molecules.

### The structural model

**Figure 7:**
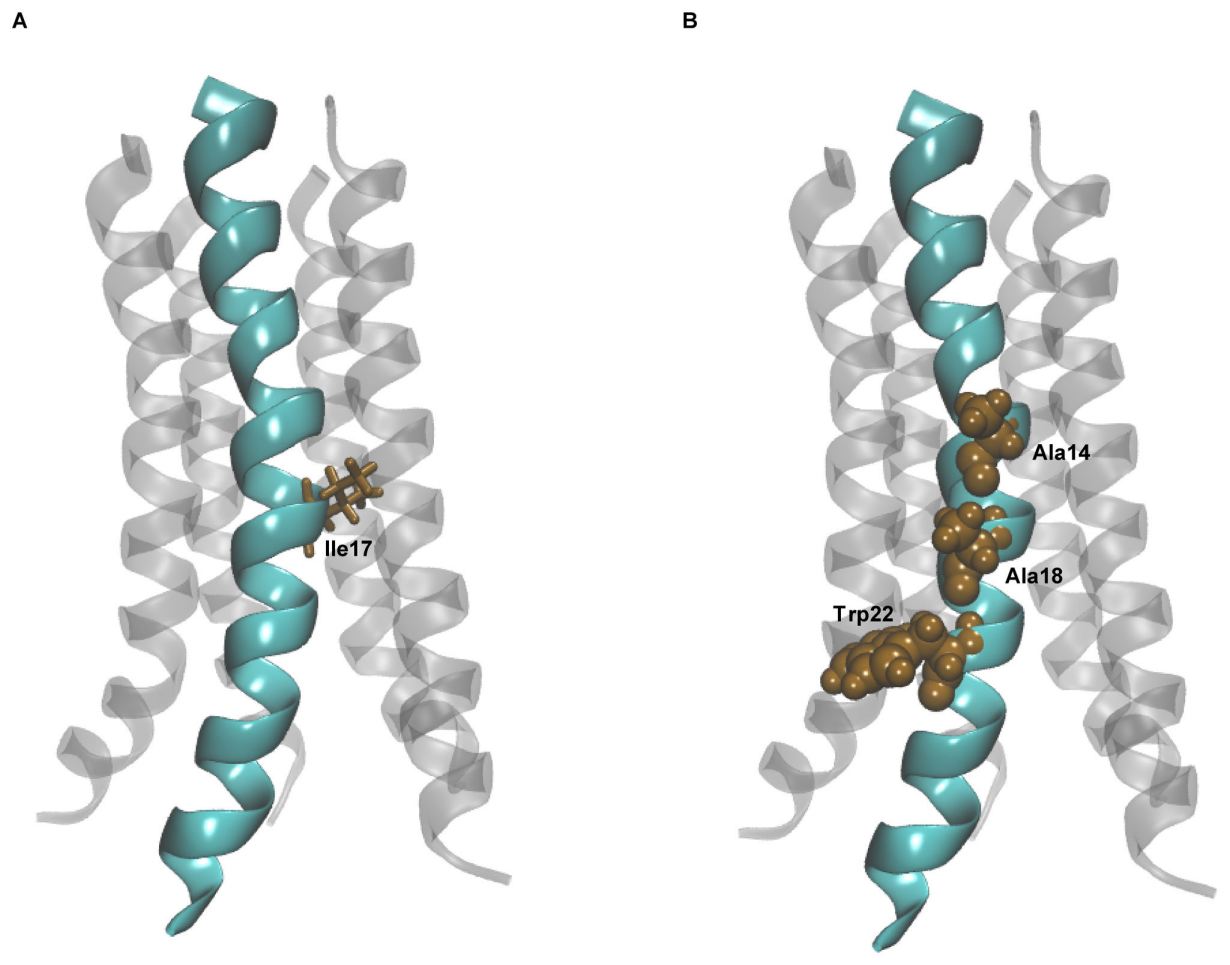
Structural features of the pentamer model. (A) Kink around the Ile17 residue in the pentamer model. (B) The three residues known to interact with tetherin shown in van der Waals representation.

In the absence of an experimentally characterized structure for the oligomeric Vpu TM domain, the proposed models offer useful insights into structural features that govern channel behavior, and into various intra- and intermolecular interactions that explain why the channel adopts a given structure. The two pentamer models discussed above are consistent with each other, with the RMSD between them differing by less than 3 Å (Figure S2 in File S1). The van der Waals interactions appear to be dominant between the helices, with the interhelical interface being formed by nonpolar residues. The importance of van der Waals forces in mediating helix-helix interactions in the membrane environment has previously been emphasized in solution NMR studies on the transmembrane peptide glycophorin A [41]. Certain structural features of the modeled structures are in remarkable agreement with available experimental data for the channel. Importantly, the kink observed in the helix around Ile17 in our model (Figure 7A and Figure S6A in File S1) is consistent with NMR studies [22]. The initial structure for the simulations had idealized α-helices without any kink, but this might be important for ion channel activity, as suggested by modeling studies on wild-type and mutant Vpu proteins [42]. Another important structural feature we observed was that the three residues known to interact with the tetherin transmembrane domain – Ala14, Ala18, and Trp22 [16] – all lie on the same face of the helix (Figure 7B and Figure S6B in File S1); this is consistent with a pentameric model generated using the structure reported in the above NMR study (PDB ID: 1PI7) [16,22]. It must be noted that the reported structure was obtained from the monomer structure followed by modeling the tetrameric and pentameric states on the basis of rotational symmetry. Such orientation of the residues is a necessary structural requirement, since, if tetherin is to bind to Vpu, it must bind to all of these residues. Moreover, the three residues lie on the exterior side of the channel, and they face the membrane rather than the pore. This has important implications. Firstly, the residues are available for binding to the tetherin transmembrane domain. Secondly, this allows the Trp22 residue to form hydrophobic contacts with lipid tails. Such hydrophobic interactions are important in stabilizing the protein in the hydrophobic lipid bilayer environment.

The Vpu channel is equally selective towards K^+^ and Na^+^, and only weakly permeable to Cl^-^ [5,7]. The highly conserved motif TTVGYGD that is seen in the selectivity filter of K^+^-specific channels [43,44] is absent in Vpu. However, because Vpu is able to discriminate cations over anions, it should have some structural motif that is responsible for this selectivity, which remains uncharacterized. Amino acids that differentially interact with ions, and thereby determine the selectivity of a channel, are likely to be charged/polar. The Vpu TM domain has five such residues – Ser23, Glu28, Tyr29, Arg30, and Lys31. While Tyr29 faces the lipid headgroup, Arg30 is stabilized via interactions with both Glu28 and the lipid headgroup. Although Lys31 faces the pore, it is unlikely that a positively charged residue will determine the selectivity of a cation-specific channel like Vpu. Moreover, Lys31 lies on the C-terminal side, where the pore is broader, and the density of pore water is high enough to shield any electrostatic effect on a permeating ion. The only other polar residue facing the pore is Ser23, and we hypothesize that this plays an important role in ion permeation. This finds support in conductance studies showing that a mutant with the serine substituted for a leucine does not exhibit any ion-conducting activity [18]. The Vpu protein from HIV-1 subtype O and P viruses, however, has a tryptophan in place of serine at position 23. Given the importance of this serine residue, Vpu from these subtypes is not expected to show ion channel activity. It is interesting to note that assays on Vpu from these subtypes report poor virus release from cells (NK and SJ; unpublished results). This further supports a role for the Vpu ion channel activity in promoting viral propagation. The occurrence of serine at position 23, and the consequent channel activity, might offer a selective advantage to M-subtype viruses, thereby accounting for the predominance of this subtype.

It is not clear whether the modeled channel is in an open or closed conformation. Certain features of the model support a closed state. The presence of a constricted region lined by hydrophobic residues is not likely to encourage the passing of an ion. Besides, the largely dehydrated nature of the pore is characteristic of closed states. However, if the modeled channel represents an open conformation, the channel is likely to show very weak ion-conducting activity, owing to the structural features described above. This possibility cannot be ruled out, since conductance studies on Vpu have shown that the channel is indeed weakly conducting [18]. It is not possible to determine whether the modeled channel is in an open state or not unless the ion-conducting activity of the channel is investigated. We are presently doing this through free energy calculations.

## Conclusion

This study comprehensively examines the possibility of the Vpu TM domain to exist in different possible oligomeric states in a hydrated lipid bilayer environment. The results suggest that the pentameric form is the most stable state, with the pentameric models possessing the symmetry that is typical of homo-oligomeric channels. The tetrameric and hexameric models, however, lose this symmetry over the course of the simulations. The major force stabilizing the pentameric form over the other forms is van der Waals interactions between adjoining helices. The pentamer is further stabilised via a salt bridge between Glu28 and Arg30, and via interactions between polar residues on the protein and lipid headgroups. These interactions are far weaker in the tetramer and hexamer, and might play a critical role in holding together the helical TM domains to the membrane. The structural features of the pentamer models from this study are able to account for much of the activity of Vpu observed in previous experimental studies. While the residues that bind to the tetherin transmembrane domain face the exterior (and are hence accessible), Ser23, which has been previously shown to be crucial in ion transport, faces the pore. The Vpu protein from O- and P-subtypes, however, is known to lack this serine at position 23, and is thus likely to have reduced ion channel activity. We hypothesize that the predominance of M-subtype viruses might be facilitated by the ability of M-subtype Vpu to conduct ions. Ion channel activity, therefore, could possibly have a role in enhancing the replication fitness of the virus. Future studies should be able to elucidate the structural and energetic factors governing ion selectivity and conductance in the channel.

## Methods

### Modeling of TM domain and replica-exchange molecular dynamics

The TM domain of Vpu is important for its ion channel activity, tetherin degradation and virus release, and hence only this domain was considered for our modeling studies. Since the TM domain is known to have a helical structure [21-23,45-47] the first 32 residues of HIV-1 NL4-3 Vpu (MVPIIVAIVALVVAIII AIVVWSIVIIEYRKI) were modeled as an idealized α-helix using the molecular modeling program SYBYL7.2 (Tripos International, St. Louis, Missouri, USA; http://www.tripos.com). While previous studies have shown residues 5 to 29 to form the TM domain [21,22,48] we have extended this by a few residues on either side to ensure that there are no destabilizing effects due to abrupt termination of the TM domain. Previous modeling studies have shown that these TM domain extensions develop α-helical conformation when modeled in a lipid bilayer environment, and that these extensions play a role in stabilizing the helix via nonbonded interactions with lipid headgroups [48]. Different oligomeric states of the TM domain were modeled using the methodology described by Bu et al. [34]. The TM domain was aligned along the z-axis, moved by a distance of 20 Å in the positive direction of the y-axis, and then given two, three, four, five, and sixfold rotational symmetries about the z-axis using the IMAGE facility in CHARMM [49,50] to give a dimer, trimer, tetramer, pentamer and hexamer, respectively. For comparison, a monomer aligned along the z-axis was also modeled. Although it is unlikely that the dimer or trimer will form the channel, these forms were modeled to examine the structural and energetic changes accompanying sequential assembly from monomer to higher oligomeric states. 

The oligomeric forms were first simulated in an implicit membrane environment. The Generalised Born model with a simple switching function (GBSW) module [33,36] was used with a surface tension coefficient of 0.03 kcal mol^-1^ Å^-2^. An implicit membrane with a hydrophobic core of thickness 35 Å was placed perpendicular to the z-axis. A smoothing region of thickness 0.5 Å was used on both sides of the hydrophobic core for a smooth transition from the hydrophobic implicit membrane to the hydophilic continuum solvent. REX/MD simulations were carried out for each oligomeric state using the CHARMM22 all-atom protein force field with CMAP corrections [51,52]. A total of eight replicas were distributed over an exponentially spaced temperature range from 300 K to 400 K. Temperatures above 400 K were not used in the study to avoid non-physical distortion of the structures. Since the oligomeric TM domains have a cylindrical geometry, a cylindrical harmonic restraint with force constant 1 kcal mol^-1^ Å^-2^ and radius 25 Å was applied, thereby limiting the drifting of the TM domains. Langevin dynamics was used with a friction coefficient of 5 ps^-1^ for heavy atoms, and exchanges were attempted every 1 ps. Simulations were run for 10 ns, with the first 1 ns being considered as the equilibration period. The last 9 ns of the trajectories from simulations at the lowest temperature (i.e., 300 K) were used for analysis. The rotational and translational entropy terms were calculated from the principal moments of inertia, while the vibrational entropy was calculated by first removing rotation and translation from all the frames, and then carrying out quasiharmonic mode analysis [53,54].

### Selection of representative structures and further equilibration

Two model structures for each of the oligomeric states (tetramer, pentamer and hexamer) were chosen for further consideration for explicit membrane simulations, details of which are given in this section. The tilt angle of the helical principal axis with respect to the membrane normal was calculated for all the conformations sampled in the last 9 ns of simulation. The range of tilt angle values that occurred most frequently was determined from a probability distribution of tilt angles. For selecting a representative structure that may further be used in extensive MD simulations, a set of structures was chosen that had tilt angles with a high probability of occurrence. From this set for each oligomeric state, the structure with the lowest molecular mechanical energy was selected as the representative structure. Each of the representative structures was then equilibrated for 10 ns in a GBSW implicit membrane with the same parameters as given above but without any constraints on any part of the protein. The temperature was kept constant at 300 K using the Nose-Hoover thermostat [55,56]. The configuration obtained after these 10 ns implicit membrane simulation of each of the three oligmeric states was taken as the other model. Since it is not straightforward to choose a reliable model without a reference structure as standard, we have considered the most sampled conformations (on the basis of tilt angle) as two different models for further calculations.

### Molecular dynamics in a fully hydrated lipid bilayer

Since the representative dimer and trimer did not form a compact structure after the above equilibration step (see Results and Discussion), only the tetramer, pentamer and hexamer were considered for further studies. To investigate the stability of these oligomeric states in a membrane-like environment, simulations were carried out in a solvated lipid bilayer. For each of these three oligomeric states, two independent simulations were carried out, one with the representative structures after the 10 ns constant temperature simulation in an implicit membrane (henceforth referred to as “Model 1”), and the other with the structures before this 10 ns simulation (“Model 2”). The protein-lipid-solvent system was set up using the CHARMM-GUI Membrane Builder [57,58]. The protein was first aligned along the z-axis and pore water was generated. A homogeneous lipid bilayer of 1-palmitoyl-2-oleoyl-sn-glycero-3-phosphocholine (POPC) was generated around all the oligomeric forms. The membrane was perpendicular to the z-axis, with its center at z = 0 Å. Bulk water of 15 Å thickness was placed above and below the membrane. Potassium and chloride ions were added to attain a salt concentration of 0.15 M KCl and a zero net charge on the system. Details about the number of each component in all the systems are shown in Table 2.

**Table 2:** Number of each component in the systems studied.

**System**	**Protein residues **	**Lipid (POPC) molecules**	**Water molecules**	**K^+^ ions**	**Cl^-^ ions**	**Total number of atoms**	**System size along x-, y-, and z-axes (in Å)**
Tetramer, Model 1	128	94	5307	13	17	30735	62 x 62 x 82
Pentamer, Model 1	160	95	5496	13	18	31984	63 x 63 x 82
Hexamer, Model 1	192	105	6848	16	22	37934	67 x 67 x 86
Tetramer, Model 2	128	85	4755	12	16	27871	60 x 60 x 80
Pentamer, Model 2	160	99	6050	15	20	34186	64 x 64 x 84
Hexamer, Model 2	192	89	5657	13	19	32211	64 x 64 x 82

The CHARMM22 all-atom protein force field including CMAP corrections [51,52], the CHARMM36 all-atom lipid force field [59], and the modified TIP3P water model [60] were used for the simulations. Periodic boundary conditions were set up, and the particle mesh Ewald method was used for calculating long-range electrostatic interactions [61]. Lennard-Jones interactions were modulated by a switching function between 10 Å and 12 Å, with all nonbonded interactions being truncated at 12 Å. The covalent bonds involving hydrogen atoms were constrained using SHAKE [62]. The system was equilibrated using the six-step equilibration scheme proposed by Jo et al. [57]. Positional harmonic restraints were applied on the ions and the heavy atoms of the protein to hold them in place during the initial equilibration simulations. Planar harmonic restraints were applied on water molecules to ensure that no water molecule entered the hydrophobic region of the membrane. Also, lipid head groups were retained close to the membrane-water interface using planar harmonic restraints. The harmonic restraints on the different components were gradually reduced during the equilibration. The first two equilibration steps were carried out in the NVT ensemble (constant volume and temperature) and the last four in the NPT ensemble (constant pressure and temperature), keeping the temperature at 303.15 K using the Nose-Hoover thermostat [55,56]. Production runs were carried out in the NPT ensemble for 10 ns with a timestep of 2 fs. The pentamer models were simulated for 30 ns to investigate the stability of the models. The molecular visualization program VMD [63] was used for rendering images and analyzing hydrogen bond interactions. Pore radius was measured using the HOLE2 program [64]. All MD simulations and other analyses were performed using the CHARMM program [49,50].

## Supporting Information

File S1 **Supporting Information**.
Table S1, Interhelical van der Waals interaction energy. Figure S1, Orientation of Ser23 before the REX/MD simulations. (A) Tetramer (B) Pentamer (C) Hexamer. Figure S2, Pair-wise RMSD values depicting how the two models vary over the trajectory. (A) Model 1 vs Model 2 (B) Model 1 vs Model 1 (C) Model 2 vs Model 2. Figure S3, Interhelical distance and protein-lipid interactions. (A) Probability distribution of interhelical distance in the explicit membrane simulations. The distance between the centres-of-mass of all helix pairs has been calculated and then averaged. (B) Hydrogen bonds between polar residues and headgroups at different intervals for Arg30 and headgroup (left panel), and Tyr30 and headgroup (right panel). Data shown is for model 2. Figure S4, Interhelical interactions. (A) Interhelical contact maps. Residue-residue distances have been averaged over time. (B) Arg30 (orange) and Glu28 (mauve) shown in licorice representation. The phosphate group (“CPK” representation) on a nearby POPC molecule (“bonds” representation) is also shown. The orientations of Arg30 and Glu28 in the tetramer, pentamer and hexamer are also shown. Figure S5, Pore profile, (A) View along the pore axis from the C-terminal showing the Ser23 residue in “licorice” representation. (B) Side view of the pentamer model showing Ser23 (“licorice” representation) and water molecules. (C) Pore radius across the axis of the pentamer model. Figure S6, Structural features of the pentamer. (A) Kink around the Ile17 residue in the pentamer model. (B) The three residues known to interact with tetherin shown in van der Waals representation.
(PDF)
