# Supplementary material for: Molecular Dynamics Simulations Reveal the HIV-1 Vpu Transmembrane Protein to Form Stable Pentamers
Source: PLoS One. 2013 Nov 6;8(11):e79779. doi: 10.1371/journal.pone.0079779 (PMC3819244; doi:10.1371/journal.pone.0079779)
Supplement: File S1 — Supporting Information. Table S1, Interhelical van der Waals interaction energy. Figure S1, Orientation of Ser23 before the REX/MD simulations. (A) Tetramer (B) Pentamer (C) Hexamer. Figure S2, Pair-wise RMSD values depicting how the two models vary over the trajectory. (A) Model 1 vs Model 2 (B) Model 1 vs Model 1 (C) Model 2 vs Model 2. Figure S3, Interhelical distance and protein-lipid interactions. (A) Probability distribution of interhelical distance in the explicit membrane simulations. The distance between the centres-of-mass of all helix pairs has been calculated and then averaged. (B) Hydrogen bonds between polar residues and headgroups at different intervals for Arg30 and headgroup (left panel), and Tyr30 and headgroup (right panel). Data shown is for model 2. Figure S4, Interhelical interactions. (A) Interhelical contact maps. Residue-residue distances have been averaged over time. (B) Arg30 (orange) and Glu28 (mauve) shown in licorice representation. The phosphate group (“CPK” representation) on a nearby POPC molecule (“bonds” representation) is also shown. The orientations of Arg30 and Glu28 in the tetramer, pentamer and hexamer are also shown. Figure S5, Pore profile, (A) View along the pore axis from the C-terminal showing the Ser23 residue in “licorice” representation. (B) Side view of the pentamer model showing Ser23 (“licorice” representation) and water molecules. (C) Pore radius across the axis of the pentamer model. Figure S6, Structural features of the pentamer. (A) Kink around the Ile17 residue in the pentamer model. (B) The three residues known to interact with tetherin shown in van der Waals representation. (PDF) [file pone.0079779.s001.pdf]

Molecular Dynamics Simulations Reveal the HIV-1 Vpu Transmembrane Protein to Form Stable Pentamers

Siladitya Padhi[1], Nabab Khan[2], Shahid Jameel[2], and U. Deva Priyakumar[1]

[1]*Centre for Computational Natural Sciences and Bioinformatics, International Institute of Information Technology, Hyderabad 500032, India*

[2]*International Centre for Genetic Engineering and Biotechnology, Aruna Asaf Ali Marg, New Delhi 110067, India*

Table S1. Interhelical van der Waals interaction energy

System	Time (ns)	Average van der Waals interaction energy per helix pair (kcal/mol)	
		**Model 1**	**Model 2**
Tetramer	0 - 2	-31.80 ± 0.05	-28.60 ± 0.06
	2 - 4	-31.16 ± 0.04	-25.93 ± 0.04
	4 - 6	-31.23 ± 0.04	-24.39 ± 0.04
	6 - 8	-33.36 ± 0.04	-23.48 ± 0.04
	8 - 10	-33.48 ± 0.05	-24.70 ± 0.04
Pentamer	0 - 2	-42.93 ± 0.04	-44.04 ± 0.05
	2 - 4	-42.55 ± 0.04	-42.37 ± 0.04
	4 - 6	-43.15 ± 0.04	-42.22 ± 0.04
	6 - 8	-43.67 ± 0.04	-41.58 ± 0.04
	8 - 10	-40.57 ± 0.04	-41.50 ± 0.04
	10 - 12	-41.86 ± 0.04	-42.18 ± 0.04
	12 - 14	-42.74 ± 0.04	-40.56 ± 0.05
	14 - 16	-42.86 ± 0.04	-40.28 ± 0.04
	16 - 18	-42.70 ± 0.04	-41.26 ± 0.04
	18 - 20	-42.35 ± 0.04	-41.09 ± 0.04
	20 - 22	-42.62 ± 0.04	-39.99 ± 0.04
	22 - 24	-43.88 ± 0.04	-40.28 ± 0.04
	24 - 26	-43.56 ± 0.04	-40.71 ± 0.04
	26 - 28	-43.21 ± 0.04	-39.91 ± 0.04
	28 - 30	-42.22 ± 0.04	-39.40 ± 0.04
Hexamer	0 - 2	-32.67 ± 0.05	-34.87 ± 0.03
	2 - 4	-30.86 ± 0.03	-34.36 ± 0.04
	4 - 6	-30.71 ± 0.04	-32.39 ± 0.04
	6 - 8	-31.44 ± 0.03	-30.81 ± 0.03
	8 - 10	-31.74 ± 0.03	-31.20 ± 0.04

The van der Waals interaction energy between all neighbouring helix pairs has been calculated and then averaged.

Figure S1. Orientation of Ser23 before the REX/MD simulations. (A) Tetramer (B) Pentamer (C) Hexamer

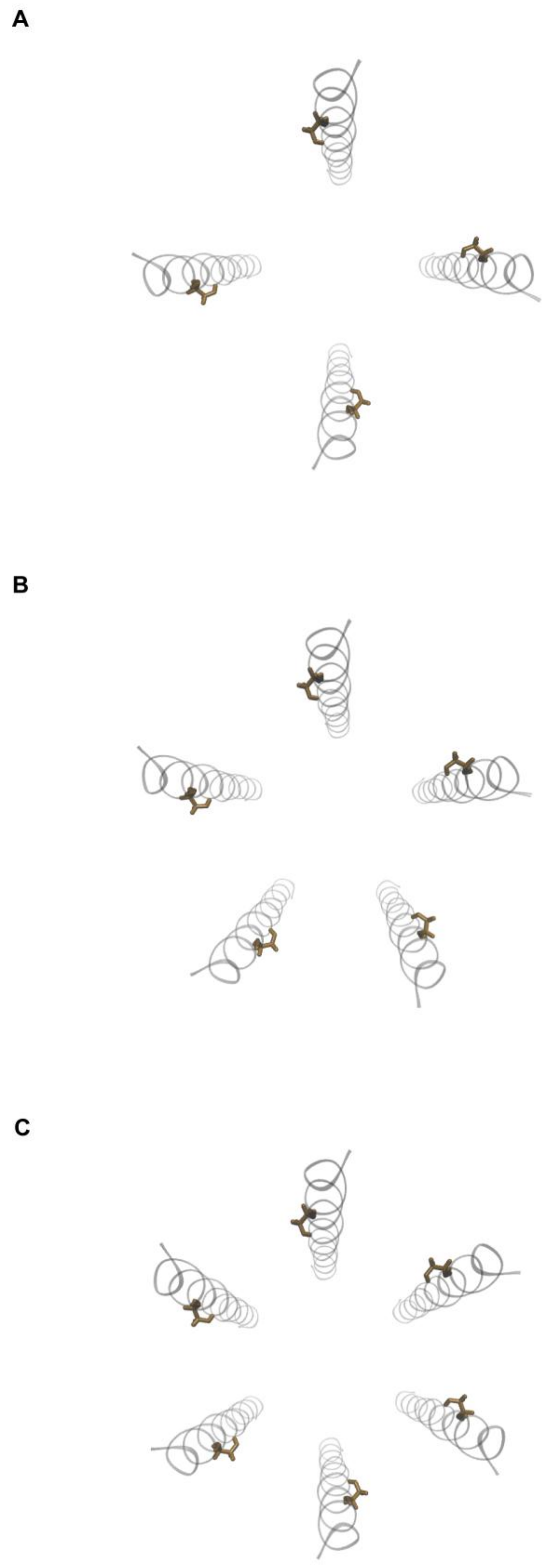

Figure S1. Orientation of Ser23 before the REX/MD simulations. (A) Tetramer (B) Pentamer (C) Hexamer

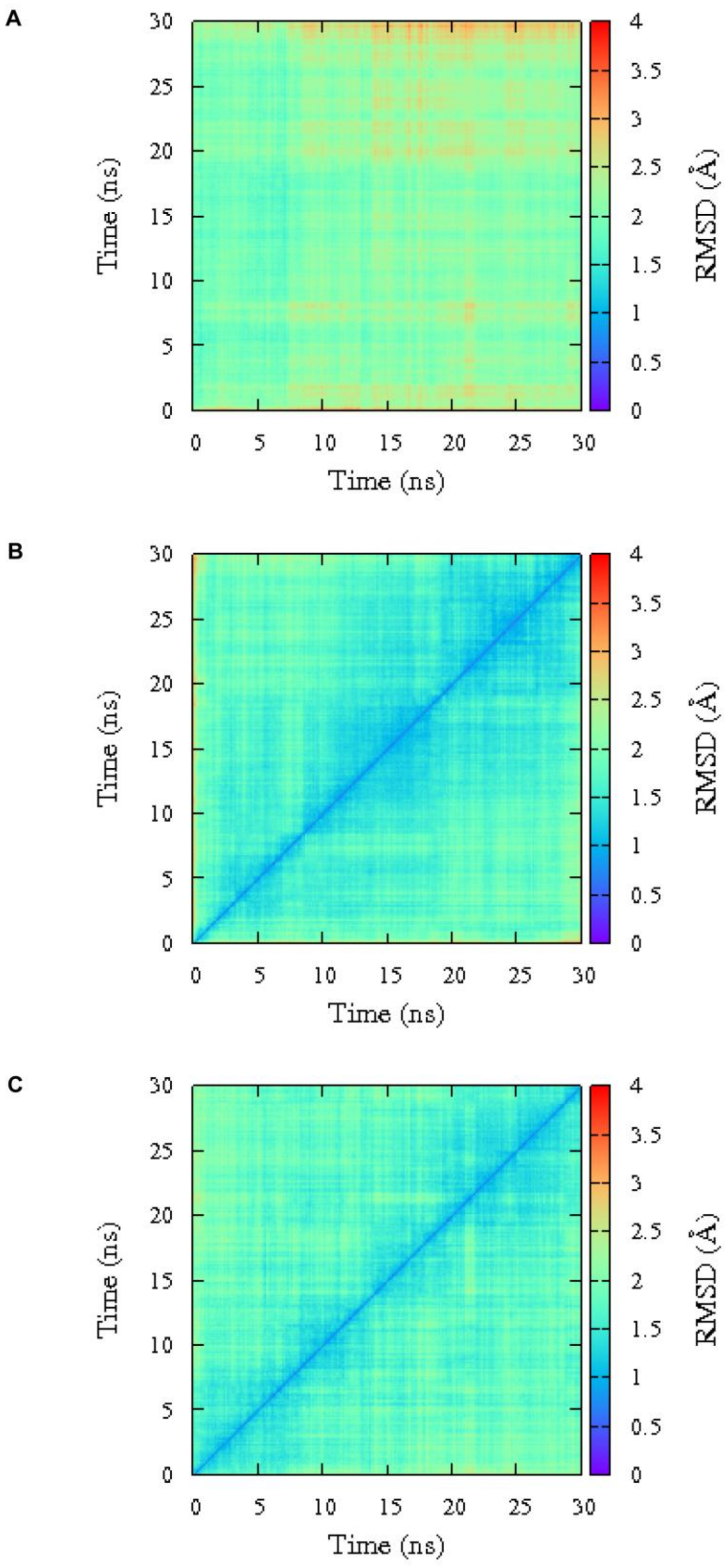

Figure S2. Pair-wise RMSD values depicting how the two models vary over the trajectory. (A) Model 1 vs Model 2 (B) Model 1 vs Model 1 (C) Model 2 vs Model 2

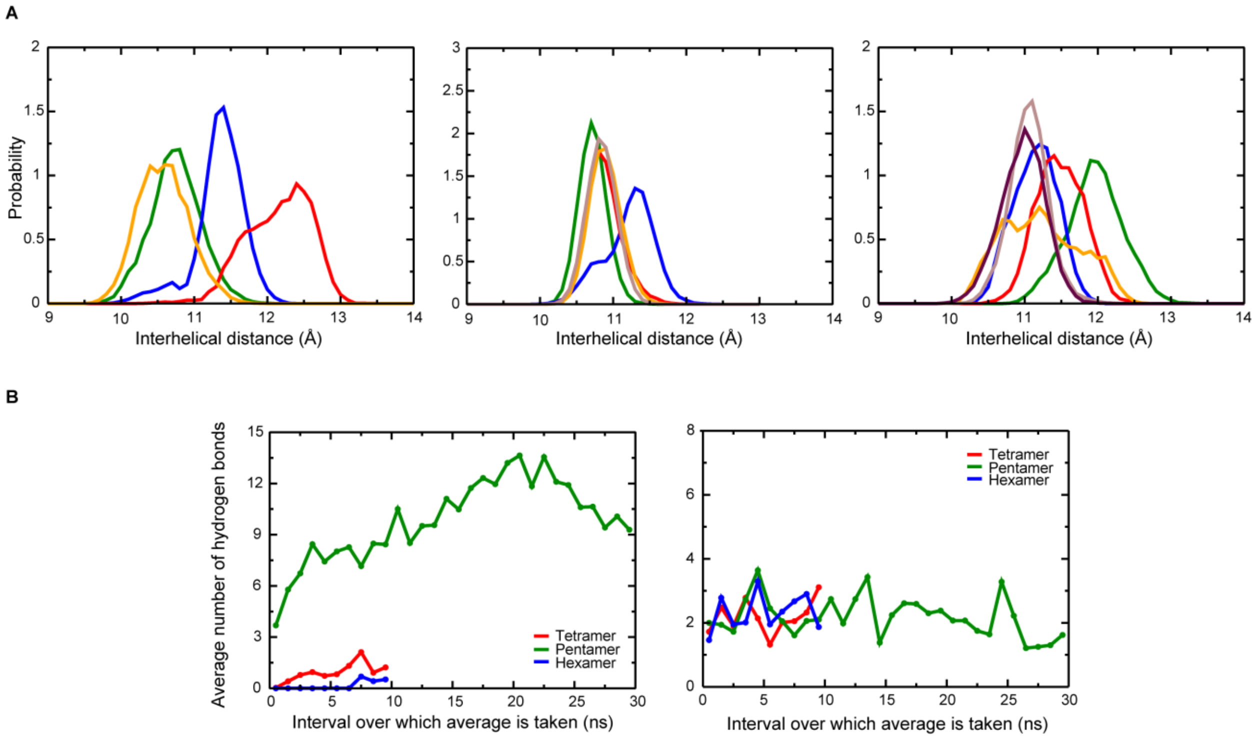

Figure S3. Interhelical distance and protein-lipid interactions. (A) Probability distribution of interhelical distance in the explicit membrane simulations. The distance between the centres-of-mass of all helix pairs has been calculated and then averaged. (B) Hydrogen bonds between polar residues and headgroups at different intervals for Arg30 and headgroup (left panel), and Tyr30 and headgroup (right panel). Data shown is for model 2.

Figures S4, S5, and S6 illustrate the structural features of Model 2.

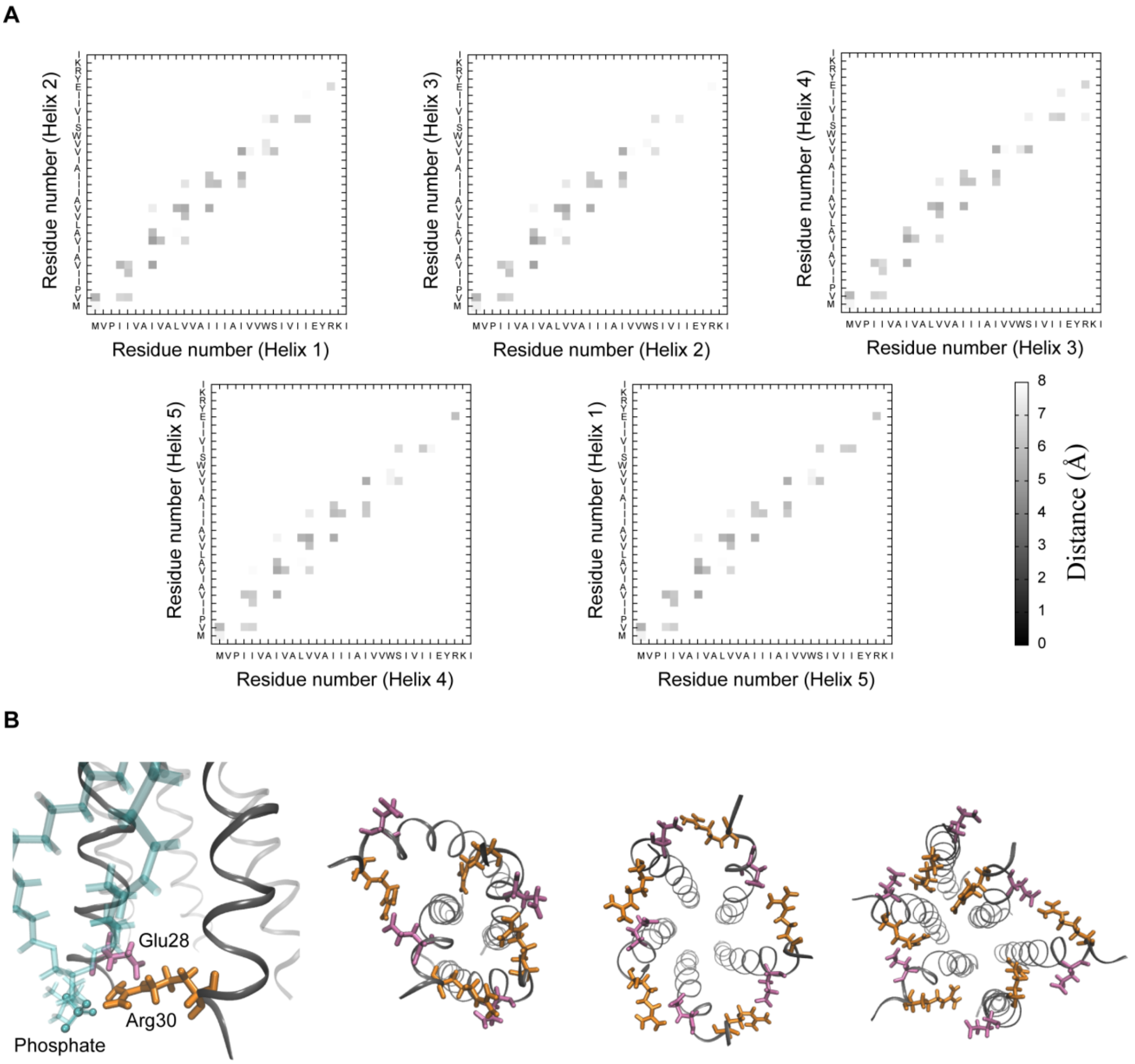

Figure S4. Interhelical interactions. (A) Interhelical contact maps. Residue-residue distances have been averaged over time. (B) Arg30 (orange) and Glu28 (mauve) shown in licorice representation. The phosphate group ("CPK" representation) on a nearby POPC molecule ("bonds" representation) is also shown. The orientations of Arg30 and Glu28 in the tetramer, pentamer and hexamer are also shown.

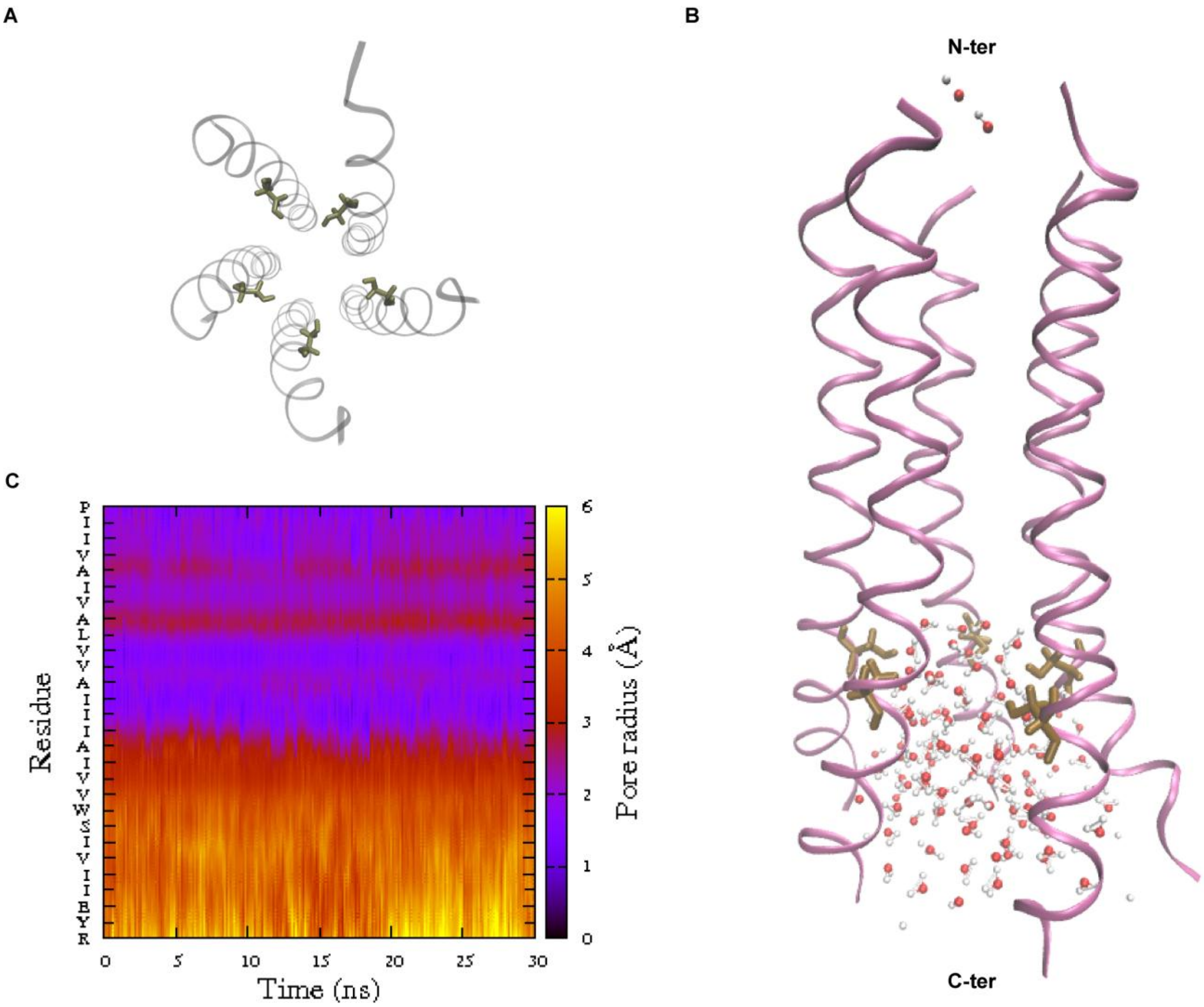

Figure S5. Pore profile. (A) View along the pore axis from the C-terminal showing the Ser23 residue in "licorice" representation. (B) Side view of the pentamer model showing Ser23 ("licorice" representation) and water molecules. (C) Pore radius across the axis of the pentamer model.

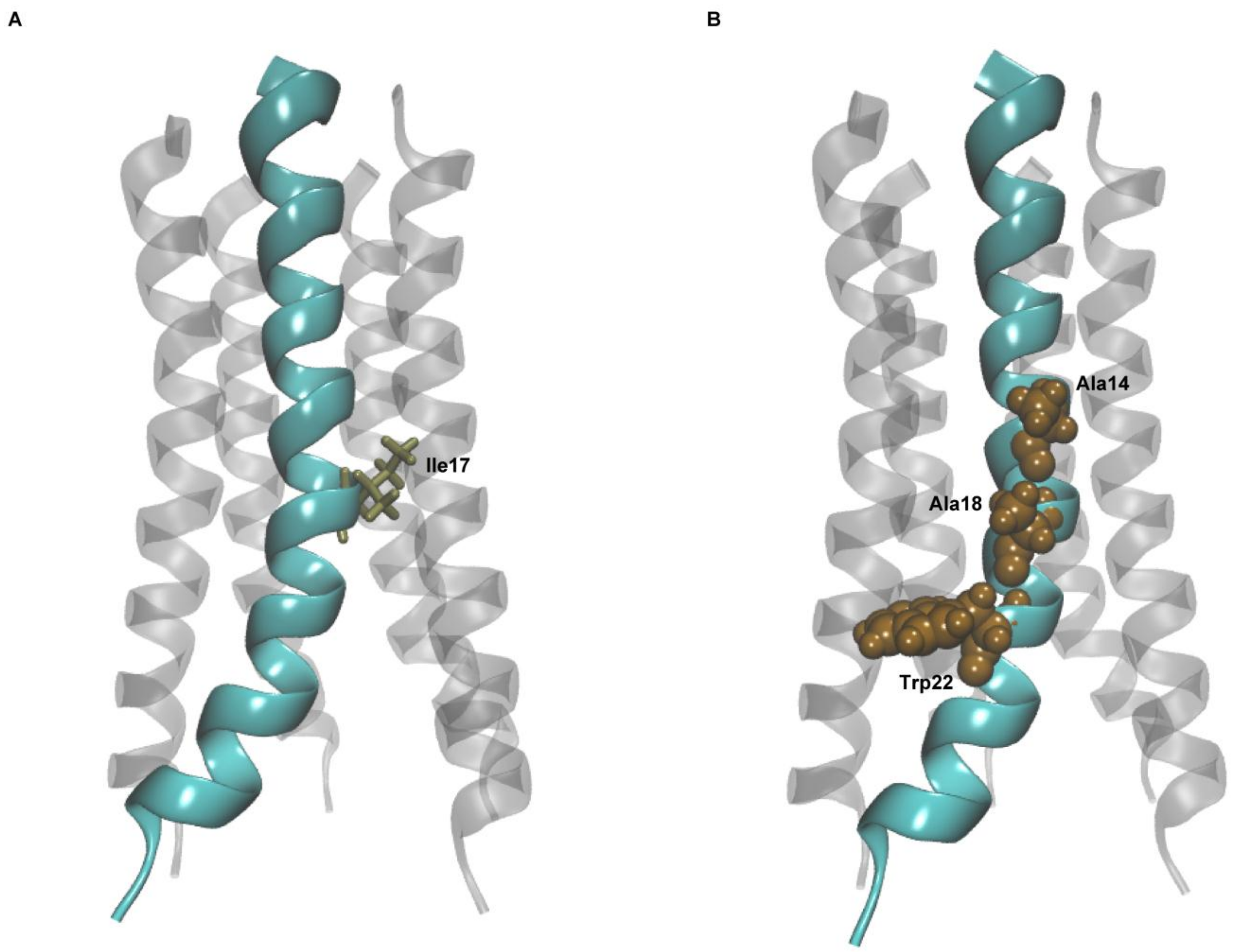

Figure S6. Structural features of the pentamer. (A) Kink around the Ile17 residue in the pentamer model. (B) The three residues known to interact with tetherin shown in van der Waals representation.
